# Old and New Symptoms

**Published:** 1887-11

**Authors:** 


OLD AND NEW SYMPTOMS.—There is perhaps no question which vexes the practitioner more than this one: Has the proper remedy been selected? Even after careful and thorough examination of the patient, and after painstaking study of the *Materia Medica*, one remains sometimes in doubt. The medicine being selected and a dose given, how shall we decide from its action whether or not it is doing service? *In acute cases* Hahnemann tells us if the medicine "excites almost no sufferings previously unfelt by the patient, produces no new symptoms, *it is* the appropriate medicine and will certainly cure the original malady." Which simply means if the patient be no worse in acute cases, whose tendency is, of course, to get worse, then the medicine is acting. Remember, then, in an acute case, where, after giving a medicine, the patient, though no better, is no worse, you must wait on the remedy. A failure to wait, in such cases, is also a failure to cure.

In chronic cases Hahnemann teaches us that where no new symptoms appear after the administration of the remedy, or where old symptoms return or are aggravated, then wait on the remedy.

The appearance of *new* symptoms in a patient after the proper administration of a remedy *never* calls for a second dose of that remedy, for such *new* symptoms show the remedy was inappropriate, was wrongly selected.

Never forget this precept: after the administration of a remedy, the reappearance (or the aggravation) of *old* symptoms is a sign that the proper drug was given, while in same case the appearance of *new* symptoms indicates an improper selection. In the former case the proper course is to wait on the remedy, in the latter case to choose another drug.

